# Ectopic Expression of the Wild Grape WRKY Transcription Factor VqWRKY52 in *Arabidopsis thaliana* Enhances Resistance to the Biotrophic Pathogen Powdery Mildew But Not to the Necrotrophic Pathogen *Botrytis cinerea*

**DOI:** 10.3389/fpls.2017.00097

**Published:** 2017-01-31

**Authors:** Xianhang Wang, Rongrong Guo, Mingxing Tu, Dejun Wang, Chunlei Guo, Ran Wan, Zhi Li, Xiping Wang

**Affiliations:** ^1^State Key Laboratory of Crop Stress Biology in Arid Areas, College of Horticulture, Northwest A&F UniversityYangling, China; ^2^Key Laboratory of Horticultural Plant Biology and Germplasm Innovation in Northwest China, Ministry of Agriculture, Northwest A&F UniversityYangling, China

**Keywords:** WRKY, salicylic acid, hypersensitive response, cell death, reactive oxygen species, Chinese wild *Vitis*

## Abstract

WRKY transcription factors are known to play important roles in plant responses to biotic stresses. We previously showed that the expression of the WRKY gene, *VqWRKY52*, from Chinese wild *Vitis quinquangularis* was strongly induced 24 h post inoculation with powdery mildew. In this study, we analyzed the expression levels of *VqWRKY52* following treatment with the defense related hormones salicylic acid (SA) and methyl jasmonate, revealing that *VqWRKY52* was strongly induced by SA but not JA. We characterized the *VqWRKY52* gene, which encodes a WRKY III gene family member, and found that ectopic expression in *Arabidopsis thaliana* enhanced resistance to powdery mildew and *Pseudomonas syringae* pv. *tomato* DC3000, but increased susceptibility to *Botrytis cinerea*, compared with wild type (WT) plants. The transgenic *A. thaliana* lines displayed strong cell death induced by the biotrophic powdery mildew pathogen, the hemibiotrophic *P. syringe* pathogen and the necrotrophic pathogen *B. cinerea*. In addition, the relative expression levels of various defense-related genes were compared between the transgenic *A. thaliana* lines and WT plants following the infection by different pathogens. Collectively, the results indicated that *VqWRKY52* plays essential roles in the SA dependent signal transduction pathway and that it can enhance the hypersensitive response cell death triggered by microbial pathogens.

## Introduction

Grapevine (*Vitis vinifera* L.) is an important fruit crop that is cultivated world-wide, however, lots of grapevine varieties are highly susceptible to infection by a large variety of pathogens. For example, powdery mildew, *Botrytis cinerea* and downy mildew all affect the growth of grapevine and reduce its fruit quality (Mzid et al., 2007). Chemicals are often used in vineyards to prevent, or limit, disease out breaks and to increase production, but this practice leads to an increase in production costs and increased risk of environmental pollution and pesticide residues, with consequent deleterious effects on human health (Zhu et al., 2012). There is therefore considerable interest in developing new cultivated grapevine varieties that are highly resistant to pathogens and that retain high quality fruit. To this end, classical crossbreeding is commonly used, but this is time consuming and the phenotypic traits of the filial generation are typically unstable, so this approach has limited potential. In contrast, the use of molecular breeding to obtain new cultivated grapevine varieties is, in many ways, easier than conventional methods. However, molecular breeding of disease-resistant grape varieties has, to date, been limited by the rudimentary understanding of the networks of defense related genes, and so identifying these in grape is an important objective.

Plants are both lack of mobile defender cells and the somatic adaptive immune system to fight against microbial pathogens that may impair plant growth and reproduction. Instead, they have developed their unique immunity mechanisms to protect themselves, which include two-branched innate immune system, namely the PAMP-triggered immunity (PTI) and effector-triggered immunity (ETI) (Dangl, 2001; Jones and Dangl, 2006). These plant immunities may share some common signaling components such as hypersensitive response (HR), reactive oxygen species (ROS), activating the expression of PATHOGENESIS-RELATED (PR) genes (Tena et al., 2011). Pathogen induced HR and ROS play important roles in plant defense. In grape, HR could limit the supply of nutrients required by the biotrophic fungus for further growth and development (Qiu et al., 2015). Meanwhile, ROS, which are generated in response to pathogen attacks, play an important role in regulating HR cell death. Besides, they are also involved in local and systemic resistance to different plant pathogens (Torres et al., 2002; Mur et al., 2008; Miller et al., 2009; Mersmann et al., 2010; Adachi et al., 2015). HR and the induction of PR proteins can be triggered by SA regulated defense mechanisms at the infection site or in distal parts of the plant, leading to the development of systemic acquired resistance (SAR) (Thomma et al., 1998; Wu et al., 2015).

Hypersensitive response and ROS are often associated with hormone regulated defense signaling pathways such as salicylic acid (SA), jasmonic acid (JA), and ethylene (ET) (Tena et al., 2011). SA signaling pathways play important roles in plant defense responses (Wu et al., 2015). In *Arabidopsis*, enhanced disease susceptibility 1 (*EDS1)* and phytoalexin deficient 4 (*PAD4)* are involved in SA signaling pathways. They play essential roles upstream of SA biosynthesis and HR (Rustérucci and Parker, 2001). *Arabidopsis* isochorismate synthase functional (*ICS1*) is involved in pathogen-induced accumulation of SA and plays essential roles in diverse stress responses (Strawn et al., 2007). In grape, *EDS1* like and *PAD4* of two grapevine species, *Vitis vinifera* cv. Cabernet Sauvignon and *V. aestivalis* cv. Norton, are associated with the SA pathway, also play important roles in grapevine defenses against powdery mildew (Gao et al., 2014).

In addition to phytohormones, diverse families of transcription factors are known to regulate plant defenses. As an example, the WRKY gene family, which is one of the largest families of transcription factors in plants (Ülker and Somssich, 2004), had been shown to modulate plant defense signaling pathways (Eulgem and Somssich, 2007; Phukan et al., 2016). *Arabidopsis thaliana WRKY18*, *WRKY40*, and *WRKY60* are all involved in responses to *Pseudomonas syringae* and *B. cinerea*, with *WRKY18* playing a more substantial part in the process (Xu et al., 2006). In addition, *A. thaliana WRKY33* is required for defense against necrotrophic fungi, and the *wrky33* mutant is highly susceptible to *B. cinerea* (Liu et al., 2015). However, loss of function of *WRKY57* has been reported to enhance host resistance to this pathogen, since *WRKY57* usually compromises *B. cinerea* resistance by competing with *WRKY33* to regulate the expression levels of jasmonate ZIM-domain (JAZ) genes, *JAZ1* and *JAZ5*, which in turn act as repressors of the JA signaling pathway (Yan and Yu, 2016).

Although little is known regarding disease resistance-related genes in grapevine, several WRKY genes have been identified that may have essential roles in defense responses. *VvWRKY1* can increase the resistance to downy mildew through jasmonic acid signaling pathway (Marchive et al., 2013). Ectopic expression of *VvWRKY2* in tobacco was reported to result in high resistance to *B. cinerea* (Mzid et al., 2007), while Chinese wild grapevine *Vitis pseudoreticulata* (*Vp) WRKY3* was shown to be specifically induced by pathogen infection, SA and ET, and its over-expression in tobacco enhanced resistance to *Ralstonia solanacearum* (Zhu et al., 2012). In addition, the expression of *VpWRKY1* and *VpWRKY2* is strongly induced by *Erysiphe necator* infection, and *VpWRKY1* or *VpWRKY2* over-expressing transgenic *A. thaliana* lines had increased resistance to powdery mildew (Li et al., 2010). The *V. pseudoreticulata EIRP1* E3 ligase has been shown to interact with *VpWRKY11* and this interaction may affect disease resistance by mediating proteolysis of the protein (Yu et al., 2013).

WRKY proteins can be phosphorylated by mitogen activated protein kinases (MAPKs) at specific sites to regulate plant defense signals (Ishihama and Yoshioka, 2012). For example, when WRKY7, WRKY8, WRKY9, and WRKY11 are phosphorylated by a MAPK, they can regulate the expression of NADPH oxidase, which triggers a ROS burst and cell death in *Nicotiana benthamiana* (Adachi et al., 2015). Other protein modifications, such as acetylation, may also affect WRKY protein function. For example, two effectors, PopP2 and AvrRps4, which are delivered by plant pathogens to suppress host defense, have evolved to block the function of WRKY transcription factors, potentially, though acetylating lysine residues in the WRKY domain, thereby affecting binding activity (Leroux et al., 2015; Sarris et al., 2015). The WRKY transcription factors can be classified into groups I–III, based on their WRKY domains and zinc-finger motifs (Eulgem et al., 2000; Wang et al., 2014). Group III genes are thought to be the most evolutionarily advanced and exhibit a high degree of adaptability (Zhang and Wang, 2005). In grapevine, 59 VvWRKY genes have been identified and classified into the three main groups (I–III) (Guo et al., 2014).

A range of wild grape genotypes have been identified in China, some of which show far greater resistance than cultivated grapevine varieties to some microbial pathogens (Wang et al., 1995). For example, Chinese wild *Vitis quinquangularis* clone Shang-24 was shown to be resistant to a number of fungal pathogens, particularly to *E. necator* (Wang et al., 1995; Wan et al., 2007). This wild grape species therefore has considerable potential as a resource for identifying disease resistance genes. In the current study, we characterized the expression of *WRKY52* from Shang-24 that had been treated with SA or methyl-jasmonate (MeJA). We also over-expressed *VqWRKY52* in *A. thaliana* and analyzed the responses of the transgenic lines to inoculation with *Golovinomyces cichoracearum*, *B. cinerea*, and *P. syringae* pv. *tomato* DC3000 (*Pst*DC3000). The results are presented and discussed in the context of a role for *VqWRKY52* in an SA dependent signal transduction pathway and in HR cell death.

## Materials and Methods

### Plant Materials, Growth Condition, and Pathogen

Chinese wild *V. quinquangularis* clone Shang-24 seedlings were grown in the grape germplasm resources orchard at the Northwest A&F University, Yangling, Shaanxi, China. Wild type (WT) *A. thaliana* (ecotype type, Columbia- 0), the *pad4* mutant and *N. benthamiana* were preserved in our lab. *A. thaliana* plants were grown under the following conditions: 21°C, 50% relative humidity and a long-day photoperiod (16 h- light/ 8 h- dark). *N. benthamiana* was grown in a growth chamber under the following conditions: 26°C, 50% relative humidity and a long-day photoperiod (16 h- light/ 8 h- dark). *G. cichoracearum* was cultured on *A. thaliana pad4* mutant plants at 21°C and a photoperiod of 16 h light/8 h dark. *Pst*DC3000 was preserved at -80°C. *B. cinerea* was maintained at 22°C on Potato Glucose Agar as described by Wan et al. (2015).

### Grape Hormone Treatments

Young leaves of 2-year-old grapes were sprayed with 100 μM SA or 50 μM MeJA (Repka et al., 2004; Wang and Li, 2006). Sterile distilled water was used as a mock control. Samples were collected at 1, 12, 24, and 48 h post treatment (hpt) and frozen at -80°C.

### Quantitative Real-Time PCR

Quantitative real-time PCR analysis was performed as previously described (Tu et al., 2016). The E.Z.N.A.^®^ Plant RNA Kit (Omega Bio-tek, USA, R6827-01) was used to extract grapevine total RNA and the RNA prep plant kit (Tiangen Biotech., China) was used to extract *A. thaliana* RNA. Prime Script TMR Tase (TaKaRa Biotechnology, Dalian, China) was used to synthesize first-strand cDNA. We used SYBR green (TaKaRa Biotechnology) and an IQ5 real-time PCR instrument (Bio-Rad, Hercules, CA, USA) to conduct quantitative real-time PCR (qRT-PCR) analysis. All of the above procedures were carried out according to the manufacturers’ instructions. *VvActin1* or *AtActin2* were used as references genes. Primers used for the qRT-PCR analyses are listed in **Supplementary Table S1**. Three biological replicates were analyzed for each experiment and three technical replicates for each biological replicate. Relative expression levels were analyzed with the IQ5 software using the Normalized Expression Method.

### Vector Construction

Total RNA extractions from leaves of *V. quinquangularis* clone Shang-24 and first-strand cDNA synthesis were performed as described above. The open reading frame (ORF) of *VqWRKY52* was amplified by PCR using the specific primers F1 (5′-**C**GGGATCCATGGAGAACATGGGAAGTTGGGAAC-3′) and R1 (5′-GGGGTACCTTAAAAGAATCCCAGGTGGTCGAAGTTA-3′). The PCR product was cloned into the pGEM-T easy vector (Promega, Madison, WI, USA), to give the construct pGEM-Teasy-*VqWRKY52*, which was then sequenced. To obtain the over-expression vector, the *VqWRKY52* ORF from pGEM-Teasy-*VqWRKY52* was inserted immediately downstream of the CaMV35S promoter in the plant over-expression vector, pCambia 2300 (Cambia, Brisbane, QLD, Australia) using the *BamH*I and *Kpn*I restriction endonucleases.

Grapevine DNA was extracted from leaves of *V. quinquangularis* clone Shang-24 as previously described (Tu et al., 2016). A 2107 bp *VqWRKY52* promoter fragment was amplified by PCR from genomic DNA using the gene specific primers F2 (5′-CCCAAGCTTCGGAATTCGCGTGATCAAAGTAATTGAGG-3′) and R2 (5′-TCCCCCGGGTTTTAAACCACCCAAAGAAGAAGAA-3′), and inserted into the binary vector pBI121 (Clontech, Palo Alto, CA, USA), to replace the CaMV 35S promoter, upstream from the β-glucuronidase (GUS) reporter gene, using the *Hind*III and *Sma*I restriction endonucleases. The resulting vector was named *pro_V qWRKY 52_*:*GUS*. pBI121 was used as a positive control and renamed *pro_35S_*:*GUS*.

### Plant Transformation

Each of the above constructs was introduced into *Agrobacterium tumefaciens* strain GV3101, and these resulting *A. tumefaciens* were used to transform *A. thaliana*, using the floral dip method (Clough and Bent, 1998). Transient expression in *N. benthamiana* was performed by infiltration as previously described (Guan et al., 2014). The infiltrated plants were maintained for an additional 3 days under the same conditions and the hormone treatments were performed as described above. For the *A. thaliana* transformation, the three T3 homozygous lines with the strongest resistance to powdery mildew (#28, #30, and #33) were selected and used for all subsequent experiments. To assess GUS activity in the transgenic *pro_V qWRKY 52_*:*GUS* plants, three independent transgenic T3 lines were analyzed.

### GUS Assays

β-Glucuronidase activity assays were performed as previously described (Jefferson et al., 1987). A vacuum was applied to the samples for 30 min prior to incubation at 37°C for 24–48 h in the staining solution [1 mM 5-bromo-4-chloro-3-indolyl-β-D-glucuronide (X-gluc; Biosynth AG), 100 mM sodium phosphate (pH 7.0), 0.5 mM K_3_Fe(CN)_6_, 0.5 mM K_4_Fe(CN)_6_, 0.1% Triton X-100 and 0.1 mM EDTA]. Chlorophyll was then cleared from the samples with 70% ethanol, and the samples were viewed under a light microscope (BX53, Olympus, Japan).

### Inoculation of *A. thaliana* with Pathogen

Four-week-old T3 transgenic and WT plants were inoculated with *G. cichoracearum*, and the number of conidiophores per colony was counted at 7 days post inoculation (dpi), as previously described (Wang et al., 2011). Samples for the expression profile analysis of defense related genes and the accumulation of O_2_^-^ were collected at 0, 24, 48, and 72 h post inoculation (hpi), samples used for analysis of fungal structures and monitoring cell death were collected at 7 dpi.

Four-week-old plants were inoculated with *Pst*DC3000 by dipping whole rosettes in *Pst*DC3000 solutions (10^8^ cfu/mL, in 10 mM Mg_2_SO_4_ supplemented with 0.025% Silwet77) as previously described (Wen et al., 2015; Guo et al., 2016). The inoculated plants were maintained under 90% relative humidity for 24 h before being moved to normal growth conditions. Samples used for morphological observation were taken at 5 dpi. Samples used for the expression analysis of defense related genes were collected at 0, 6, 12, and 24 hpi. Leaves inoculated with *Pst*DC3000 by infiltration (Varet et al., 2003) were used to monitor the bacterial growth at 3 dpi, and the detection of cell death was performed at 0, 24, 48, and 72 hpi, while accumulation of O_2_^-^ and H_2_O_2_ was measured at 0 and 72 hpi.

The *B. cinerea* conidial suspension (1.5 × 10^6^ conidia/ml) used for inoculation was prepared as previously described (Wan et al., 2015). Detached leaves were used for morphological observation and the lesion diameter analysis, which were performed by droplet inoculating with 10 μL of the conidial suspension, as previously described (Guo et al., 2016). Samples used for morphological observation and lesion diameters analysis were photographed and measured at 3 dpi. Adult plants were inoculated by spraying, as previously described (Wan et al., 2015), and were then used for analyzing the expression of defense related genes at 0, 12, 24, and 48 hpi. Cell death was measured at 0, 24, 48, and 72 hpi and the accumulation of O_2_^-^ and H_2_O_2_ was measured at 0 and 48 hpi.

### ROS Levels and Cell Death Assay

Cell death and the fungal structures were visualized by staining with trypan blue as previously described (Vogel and Somerville, 2000). Diaminobenzidine (DAB) staining was used to detect the accumulation of H_2_O_2_ (Fryer, 2002), and nitro blue tetrazolium (NBT) staining was used to detect the accumulation of O_2_^-^ (Kim et al., 2011), as previously described. All samples were imaged with a light microscope (Olympus, Japan). At least six leaves were used for each independent experiment and three biological replicates were analyzed.

### Statistical Analysis

Data analysis and plotting were performed using Microsoft Excel (Microsoft Corporation, USA) and Sigma plot (v. 10.0, Systat, Inc., Point Richmond, CA, USA). Significant differences were assessed through paired *t*-test using the SPSS Statistics software (IBM China Company, Ltd, Beijing, China) as previously described (Tu et al., 2016). All experiments were performed using three biological replicates, with each biological replicate having three technical replicates.

### Accession Numbers

The accession numbers of the genes used in this paper are found in The *Arabidopsis* Information Resource^1^ and the grape genome Sequence^2^: *AtActin2* (*AT3G18780*), *AtEDS1 (AT3G48090)*, *AtPR1 (AT2G14610), AtPR2 (AT3G57260), AtPR5 (AT1G75040), AtPDF1.2 (At5g44420), AtICS1 (At1g74710), VvActin1 (AY680701), VqWRKY52 (KY411919).*

## Results

### *VqWRKY52* Expression is Induced by SA Treatment

**FIGURE 1:**
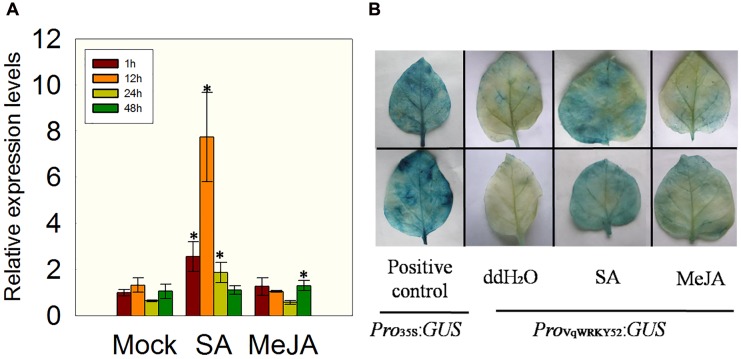
**Expression of *VqWRKY52* following salicylic acid (SA) and methyl jasmonate (MeJA) treatments. (**A**)** Relative expression levels in *Vitis pseudoreticulata* by qRT-PCR. (**B**) Analysis of *VqWRKY52* promoter activity in *Nicotiana benthamiana*. Bars represent the mean ± SD from three independent experiments. Asterisks indicate statistical significance between treatment and mock (^∗^0.01 < *P* < 0.05, Student’s *t*-test).

In previous studies, a transcriptome analysis of Chinese wild *V. quinquangularis* clone Shang-24 at different time points after inoculation with *E. necator* indicated that *VqWRKY52* was highly induced by this treatment (Jiao et al., 2015). To identify which hormones could affect the expression of *VqWRKY52*, we measured its expression levels in *V. quinquangularis* clone Shang-24 at 1, 12, 24, and 48 h post treatment with SA or MeJA. We observed that the expression of *VqWRKY52* was strongly induced by SA treatment, but not by MeJA, at 12 h post treatment, compared to the mock treatment, followed by a decrease at 24 h compared with the expression levels at 1 and 12 h (**Figure 1A**).

To validate this result, a 2107 bp *VqWRKY52* promoter fragment was fused to the *GUS* reporter gene and transiently expressed in *N. benthamiana*, using a *Pro_35S_*:*GUS* construct as a positive control. The transiently expressing leaves were treated with SA, MeJA or a negative ddH_2_O control and then subjected to GUS staining. Leaves expressing the *Pro_35S_*:*GUS* construct showed strong GUS activity, while little activity was detected in the leaves transiently expressing the *Pro_V qWRKY 52_*:*GUS* construct. However, when *Pro_V qWRKY 52_*:*GUS* expressing leaves were treated with SA, high GUS activity levels were observed, while only low levels of activity were detected in MeJA treated leaves (**Figure 1B**). This was consistent with the expression results presented in **Figure 1A**.

### Cloning and Sequence Analysis of *VqWRKY52*

Gene specific primers were designed according to the *VvWRKY52* (*GSVIVT01028718001*) cDNA sequence from the Grape Genome Database (12×^2^) and used to amplify the *VqWRKY52* ORF. The *VqWRKY52* coding sequence (CDS) is 1092 bp, encoding a 364 amino acid protein, and the nucleotide sequence had 99.27% identity to the *V. vinifera* homolog, with only eight single nucleotide polymorphisms (SNPs) found between the CDS from the two grape genotypes (**Supplementary Figure S1**). The corresponding deduced amino acid sequences shared 99.18% identity (**Supplementary Figure S2**).

### *VqWRKY52* Expression Patterns

**FIGURE 2:**
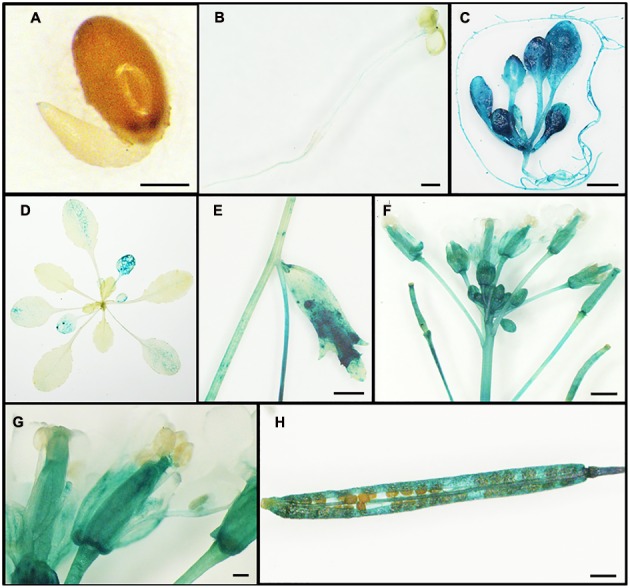
**Expression of *VqWRKY52* in *Arabidopsis thaliana.*** T3 homozygous *Pro_V qWRKY 52_*:*GUS* plants were stained with 5-bromo-4-chloro-3-indolyl-β-D-glucuronic acid at different growth stages. (**A**) Mature embryos were cultivated on Murashige-Skoog (MS) basal medium for 24 h. Scale bar = 200 μm. **(B)** 5-day-old seedling. Scale bar = 500 μm. **(C)** 2-week-old plant. Scale bar = 2 mm. **(D)** 3-week-old plant. **(E)** Stalk. Scale bar = 2 mm. **(F)** Inflorescence. Scale bar = 1 mm. **(G)** Flower. Scale bar = 200 μm. **(H)** Silique. Scale bar = 1 mm.

To further understand the temporal and spatial expression profile of *VqWRKY52*, transgenic *A. thaliana* lines constitutively expressing the *Pro_V qWRKY 52_*:*GUS* construct were generated. Plants from the T3 generation were stained for *GUS* activity and while not activity was detected at the germination stage (**Figure 2A**), during early seedling growth, low GUS activity was observed in the cotyledon tips and roots (**Figure 2B**). Two-week-old plants grown on Murashige-Skoog (MS) basal medium showed strong *GUS* activity in all organs, but especially in leaves (**Figure 2C**). Aging leaves of 3-week-old plants in soil showed more *GUS* activity than the young leaves (**Figure 2D**), and strong *GUS* activity was observed in flowers and siliques, but not in the seeds and anthers (**Figures 2E–H**).

### Over-Expressing *VqWRKY52* in *A. thaliana* Enhances Resistance to Powdery Mildew

**FIGURE 3:**
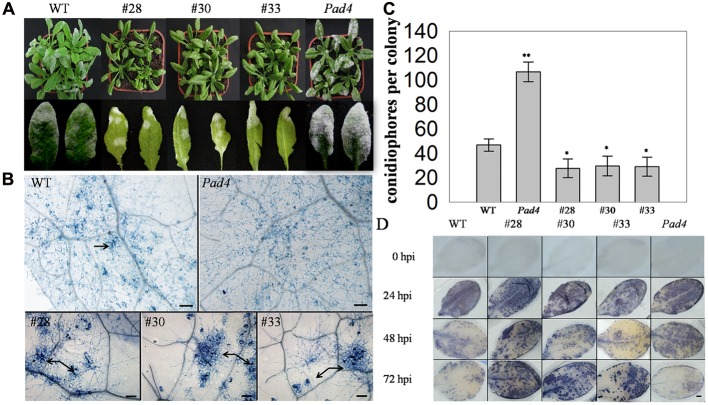
**Phenotype of *VqWRKY52* over-expressing lines inoculated with powdery mildew (*G. cichoracearum*).** **(A)** Over-expressing lines (#28, #30, #33), *pad4* mutant plants and wild type (WT) plants were infected with powdery mildew. Plants were photographed 7 days post-inoculation (dpi). **(B)** Fungal structures and plant cell death were stained with trypan blue at 7 dpi. The cell death induced by *G. cichoracearum* colonies is was highlighted by the arrow. Scale bar = 100 μm. **(C)** The accumulation of O_2_^-^ in different plants at 0, 24, 48, and 72 h post-inoculation (hpi). O_2_^-^ was visualized with nitro blue tetrazolium (NBT). Scale bar = 2 cm. **(D)** Quantitative analysis of conidiophore formation on different plants at 7 dpi. Bars represent the mean ± SD from three independent experiments. Asterisks indicate statistical significance between the over-expressing lines, the *pad4* mutant plants and WT plants (^∗^0.01 < *P* < 0.05, ^∗∗^*P* < 0.01, Student’s *t-*test).

To further investigate the putative function of *VqWRKY52* in defense process, three T3 generation transgenic *A. thaliana* lines expressing *VqWRKY52* (**Supplementary Figure S3**), together with WT, and the *A. thaliana pad4* mutant, which is susceptible to powdery mildew, were inoculated with *G. cichoracearum.* Over-expressing lines showed enhanced resistance to *G. cichoracearum* at 7 dpi (**Figure 3A**) and showed a large number of dead cells, while minimal cell death was observed in the WT plants and no obvious cell death occurred in the *pad4* mutant (**Figure 3B**). The *G. cichoracearum* colonies growing on the *pad4* mutant were the largest, followed by those on the WT plant, while the three over-expressing lines had the smallest colonies. This correlated with the extent of the cell death triggered by *G. cichoracearum* that was observed at the infection site, which is consistent with cell death restricting fungal growth (**Figure 3B**). We also counted the number of conidiophores per colony from the five different genotypes (**Figure 3C**) and determined that the three transgenic lines had a significantly fewer than the WT plants.

Since the accumulation of the superoxide anion (O_2_^-^) is associated with cell death (Yi et al., 2011), we measured O_2_^-^ levels in the different genotypes at 0, 24, 48, and 72 hpi. A significant difference was observed between WT, *pad4*, and the three transgenic lines at 48, 72 hpi, with high levels in the latter. Interestingly, almost all of the leaves from the tested lines accumulated O_2_^-^ at 24 hpi, while the mottled leaves with spots of O_2_^-^ accumulation were appeared at 48 and 72 hpi (**Figure 3D**).

### The Expression of Defense Related Genes Post Inoculation with *G. cichoracearum*

**FIGURE 4:**
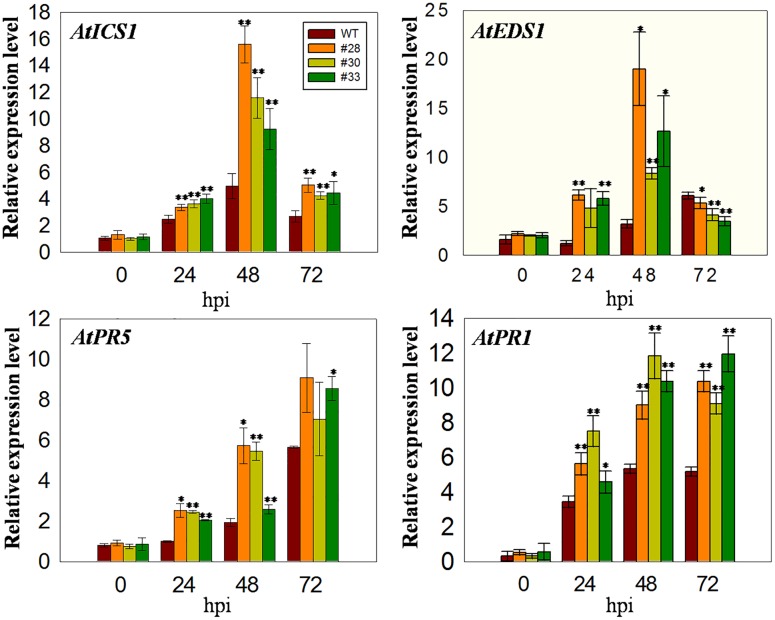
**Quantitative analysis of the expression of defense-related genes in *VqWRKY52* over-expressing lines and WT plants following *G. cichoracearum* infection.** Relative expression levels of *AtICS1, AtEDS1, AtPR1*, and *AtPR5* were analyzed using qRT-PCR. Bars represent the mean ± SD from three independent experiments. Asterisks indicate statistical significance between the over-expressing lines and WT plants (^∗^0.01 < *P* < 0.05, ^∗∗^*P* < 0.01, Student’s *t-*test).

Since *VqWRKY52* expression was induced by SA (**Figures 1A,B**), we hypothesized that it operates via SA mediated signaling pathways and the expression levels of marker genes involved in SA signaling pathways in *Arabidopsis* will be affected in three over-expressing lines. To test this, we measured the expression of four marker genes. The expression of *AtICS1*, which is involved in SA biosynthesis and affects SA accumulation (Strawn et al., 2007), was higher in the over-expressing lines than in the WT at all three time point post-inoculation, showing an initial increase at 24 hpi, peaking at 48 hpi and declining again at 72 hpi. *AtEDS1* is involved in the SA related signaling pathway and plays essential roles upstream of SA biosynthesis (Rustérucci and Parker, 2001). The expression of this gene was similar to *AtICS1* at 24 and 48 hpi; however, its expression levels were lower in the over-expressing lines than in the WT at 72 hpi. In addition, the expression of *AtPR1* and *AtPR5* increased at 24, 48, and 72 hpi compared to 0 hpi, with expression being higher in the three over-expression lines than in the WT (**Figure 4**).

### Over-Expressing *VqWRKY52* in *A. thaliana* Enhances Resistance to *Pseudomonas*

**FIGURE 5:**
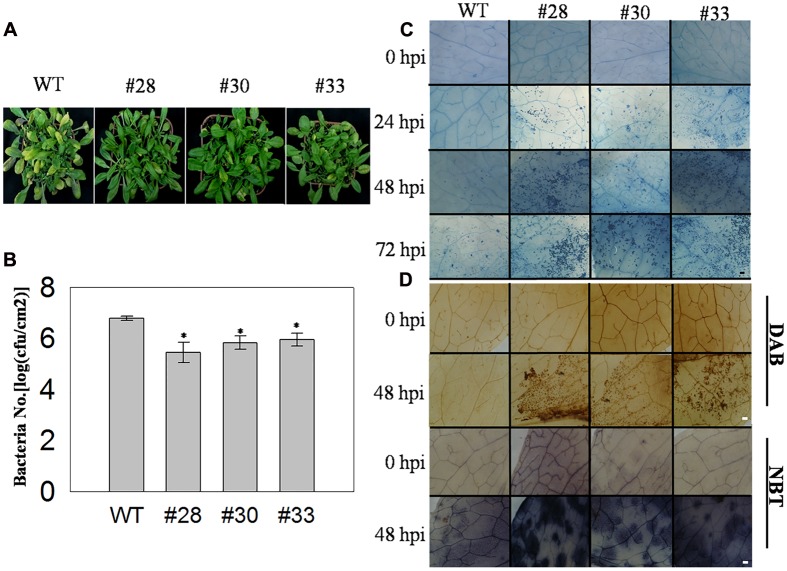
**The response of *VqWRKY52* over-expressing lines and WT to *Pst* DC3000 infection.** **(A)** Infected leaves from over the overexpression lines and WT plants were photographed 5 dpi. **(B)** The number of bacterial cells in the leaves was determined at 3 dpi. **(C)** The plant cell death induced by *Pst*DC3000 at 0, 24, 48, and 72 hpi was visualized by staining with trypan blue. Scale bar = 200 μm. **(D)** H_2_O_2_ and O_2_^-^ accumulation in the overexpression lines and WT plants at 0 and 48 hpi. H_2_O_2_ were stained with DAB and O_2_^-^ with NBT in WT and overexpression plants. The experiment was repeated three times, and at least six leaves were used in each independent experiment. Scale bar = 200 μm. The experiments have three independent biological replicates, and for each biological replicate three technical replicates were analyzed. Data represent mean values ± SD from three independent experiments. Asterisks indicate statistical significance between the overexpression lines and WT (^∗^0.01 < *P* < 0.05, Student’s *t-*test).

To examine the association between *VqWRKY52* and responses to infection by *Pst*DC3000, three T3 generation transgenic lines over-expressing *VqWRKY52* and WT plants were inoculated with *Pst*DC3000 and examined at 5 dpi. The three transgenic lines showed increased resistance to *PstDC3000*, based on less severe disease symptoms and fewer diseased leaves than the WT plants (**Figure 5A**). We also measured the growth of *Pst*DC3000 by counting the bacterial numbers per unit leaf area at 3 dpi. As shown in **Figure 5B**, we observed less growth in the three over-expressing lines than in WT plants, which suggested *VqWRKY52* mediated suppression of bacterial growth. The transgenic lines showed enhanced resistance to *Pst*DC3000 and strongly induced cell death. Specifically, no cell death was detected in the three transgenic lines and WT plants at 0 hpi, and less cell death was observed in the transgenic lines than in the WT plants at 24 hpi. Strong cell death was apparent at 48 hpi and was enhanced in the overexpression lines at 72 hpi. At this time point less cell death was detected in the WT plants to an extent that was similar to that observed in the transgenic lines at 24 hpi (**Figure 5C**). Strong cell death may be associated with a burst of ROS production and so we examined the accumulation of O_2_^-^ and H_2_O_2_ at 72 hpi, where more cell death was detected in the transgenic lines than in the WT plants. We found a larger accumulation of O_2_^-^ and H_2_O_2_ in the transgenic lines than in the WT plants (**Figure 5D**).

### The Expression Levels of Defense Related Genes Post Inoculation with *Pst*DC3000

**FIGURE 6:**
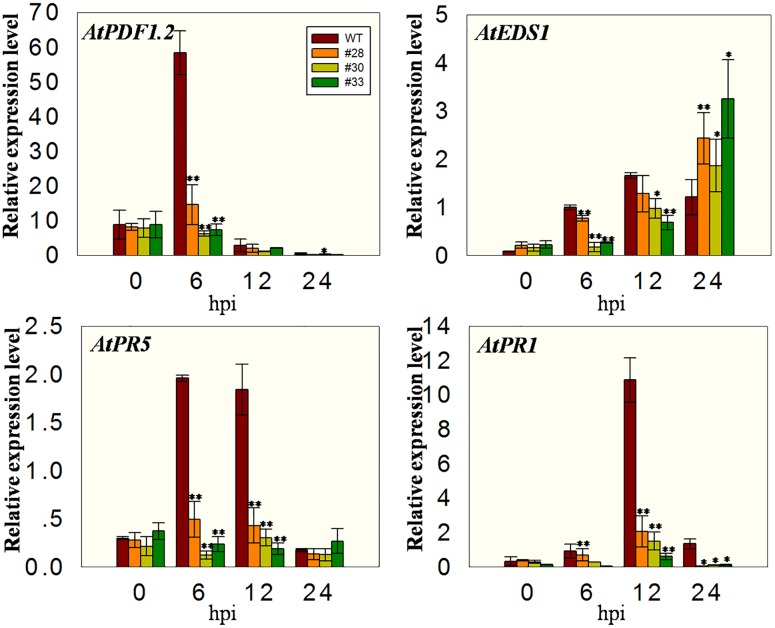
**Quantitative analysis of defense-related genes in *VqWRKY52* over-expressing lines and WT plants following *Pst*DC3000 inoculation.** Relative expression levels of *AtEDS1, AtPR1, AtPR5*, and *AtPDF1.2* were analyzed using qRT-PCR. Bars represent the mean ± SD of three independent experiments. Asterisks indicate statistical significance between transgenic lines and WT (^∗^0.01 < *P* < 0.05, ^∗∗^*P* < 0.01, Student’s *t-*test).

Since cell death induced by *Pst*DC3000 first appeared at 24 hpi in the three over-expressing lines and WT plants, we analyzed the expression levels of defense related genes at earlier times points, specifically 0, 6, 12, and 24 hpi. The expression of *AtPR1*, *AtPR5*, and *AtEDS1* was induced in WT plants, but inhibited in the transgenic lines to varying degrees, which is the opposite of their expression pattern following the powdery mildew infection. This was also true for the *AtPDF1.2* gene, which is associated with the MeJA signaling (Xu et al., 2006) (**Figure 6**).

### Over-Expressing *VqWRKY52* in *A. thaliana* Enhances Susceptibility to *B. cinerea*

**FIGURE 7:**
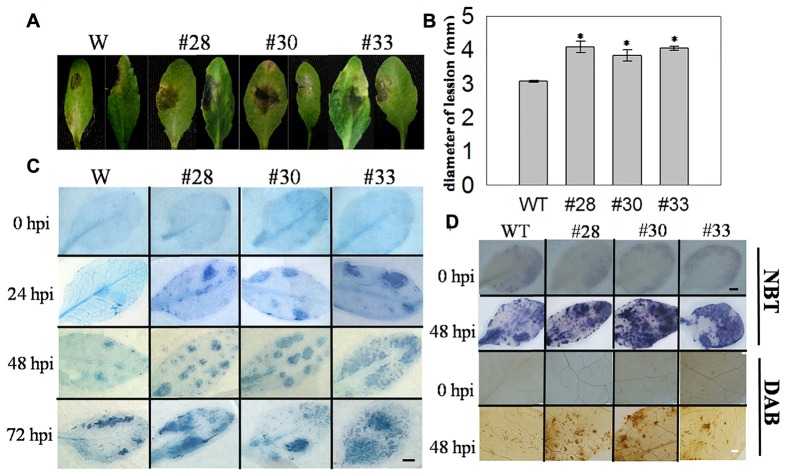
**The responses of *VqWRKY52* over-expressing lines and WT to *Botrytis cinerea* inoculation.** **(A)** Infected leaves from transgenic lines and WT plants were photographed 3 dpi. **(B)** The average diameters of lesions of infected leaves at 3 dpi. **(C)** The plant cell death induced by *B. cinerea* at 0, 24, 48, and 72 hpi were stained with trypan blue. Scale bar = 2 cm. **(D)** H_2_O_2_ and O_2_^-^ accumulation in transgenic lines and WT at 0 and 48 hpi. H_2_O_2_ were stained with DAB and O_2_^-^ with NBT in WT and over-expressing plants. The experiment was repeated three times, at least six leaves were used in each independent experiment. Black bar = 2 cm; White bar = 200 μm. Data represent mean values ± SD from three independent experiments. Asterisks indicate statistical significance between transgenic lines and WT (^∗^0.01 < *P* < 0.05, Student’s *t-*test).

Cell death induced by pathogens is important in the resistance to biotrophic pathogens, and we also wanted to determine whether this was also true for necrotrophic pathogens, such as *B. cinerea*. We observed the detached leaves of three overexpression lines and WT plants that had been inoculated with *B. cinerea* at 3 dpi and measured the lesion diameters of infected leaves, which were larger in the overexpression lines (**Figures 7A,B**). Subsequently, three plants from each genotype were incubated with *B. cinerea* by spraying, and cell death was detected at 0, 24, 48, and 72 hpi, with ROS staining at 0 and 48 hpi. Compared with the WT plants, cell death was more extensive in the transgenic lines at 24, 48, and 72 hpi, but was not observed at 0 hpi (**Figure 7C**). Minimal O_2_^-^ and H_2_O_2_ accumulation was observed at 0 hpi in the *VqWRKY52* over-expressing lines and WT plants, while higher levels were detected in the three over-expressing lines than in WT plants at 48 hpi (**Figure 7D**).

### Relative Expression Levels of Defense Related Genes Post Inoculation with *B. cinerea*

**FIGURE 8:**
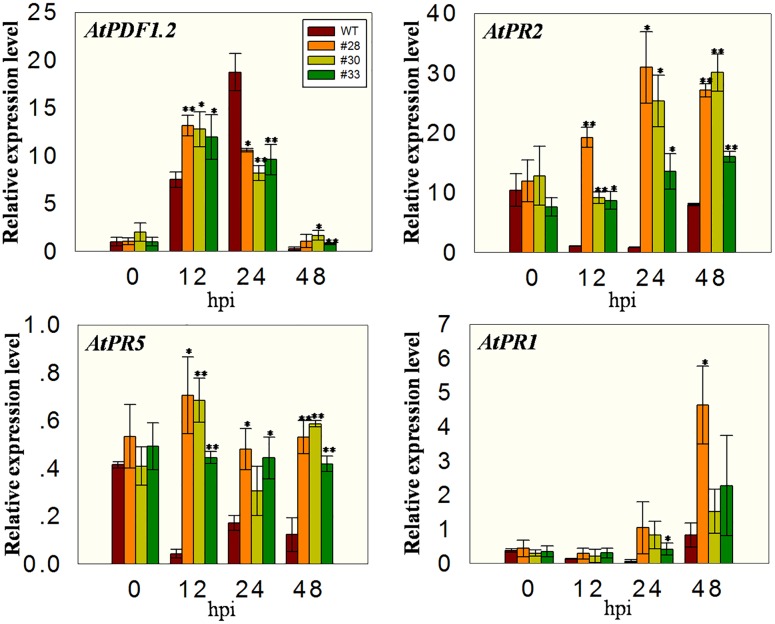
**Quantitative analysis of defense-related genes in *VqWRKY52* over-expressing lines and WT plants following *B. cinerea* inoculation.** Relative expression levels of *AtPR1, AtPR2, AtPR5*, and *AtPDF1.2* were analyzed using qRT-PCR. Bars represent the mean ± SD from three independent experiments. Asterisks indicate statistical significance between transgenic lines and WT (^∗^0.01 < *P* < 0.05, ^∗∗^*P* < 0.01, Student’s *t-*test).

To investigate whether the over-expressing lines had altered expression of defense-related genes, we measured the transcript levels of *AtPR1*, *AtPR2*, *AtPR5*, and *AtPDF1.2* at different time points after *B. cinerea* inoculation. All plants showed low expression of *AtPDF1.2* before inoculation and an induction of expression at 12 hpi, with the three transgenic lines having higher levels than the WT plants. At 24 hpi, the expression in WT plants was high, whereas it declined in the three transgenic lines such that the WT plants had the highest levels. Low *AtPDF1.2* expression levels were detected at 48 hpi in all the plants, while the expression of *AtPR2* and *AtPR5* was low at 12, 24, and 48 hpi in the WT plants compared with 0 hpi, but were significantly higher in the overexpression lines than in the WT at all three time point, indicating less inhibition. Although the expression of *AtPR1* was more highly induced in the overexpression lines than in the WT plants following the incubation with powdery mildew, no significant differences were observed in these plants following incubation with *B. cinerea* (**Figure 8**).

## Discussion

The WRKY transcription factor family is one of the largest in plants and members play important roles in signaling networks that regulate many plant processes, including defense signaling (Rushton et al., 2010). In grape, 59 grape WRKY genes (VvWRKY) have been identified and classified into three main groups (I–III) (Guo et al., 2014). Recent studies have shown that the WRKY domain of the NOD-like receptor *RRS1-R*, which is blocked by the pathogen effectors *PopP2* and *AvrRps4*, belong to Group III (Leroux et al., 2015; Sarris et al., 2015), indicating the importance of the Group III genes in plant defense. The *VqWRKY52* gene belongs to Group III (Guo et al., 2014). An earlier analysis revealed that *VqWRKY52* is strongly induced post inoculation with *E. necator* (Guo et al., 2014). Here, we observed a strong increase in expression by SA treatment, suggesting a role for *VqWRKY52* in disease resistance. So we tested the responses to powdery mildew, *Pst*DC3000 and *B. cinerea* in *A. thaliana VqWRKY52* over-expressing lines and WT plant.

It is known that SA signaling plays important roles in plant defense. Previous studies of *WRKY70*, a member of the group III in *A. thaliana*, indicated that its expression was strongly induced by SA (Li et al., 2004). Furthermore, *WRKY70* over-expressing lines showed an increased resistance to the biotroph fungi *Erysiphe cichoracearum* (*Eci*), but decreased resistance to a necrotroph fungi (Li et al., 2006). In this current study, the expression of *VqWRKY52* in *V. quinquangular* was induced by SA treatment but not by MeJA (**Figure 1A**), suggesting that it may be involved in the SA related defense signaling. The expression analysis of *VqWRKY52* promoter in *N. benthamiana* together with the enhanced expression of *AtEDS1* and *AtICS1* post inoculation of *G. cichoracearum* were consistent with it being involved in the SA signaling pathway but not in JA mediated responses (**Figure 1B**), further indicating that it plays a role in resistance to biotrophs.

When WT plants, the *pad4* mutant and the *VqWRKY52* over-expressing lines were incubated with *G. cichoracearum*, the latter showed greater resistance than the others (**Figure 3**). Interestingly, *G. cichoracearum* inoculation induced strong cell death and, accordingly, fungal growth was inhibited to a greater degree, in the three over-expressing lines (**Figures 3A,B**). Pathogen-induced HR cell death is thought to be an important plant defense response (Wang et al., 2011), and many genes involved in cell death and plant defense have been identified. These genes can be grouped into two classes: genes involved in spontaneous cell death and genes involved in enhanced pathogen-induced cell death. The *A. thaliana enhanced disease resistance 1* (*EDR1*) is a suppressor of plant defenses, and associated with pathogen-induced cell death, and the *edr1* mutant shows enhanced disease resistance and powdery mildew-induced cell death (Serrano et al., 2014). This suggests an important role for *VqWRKY52* in resistance of powdery mildew associated with pathogen-induced cell death.

*PAD4* is also involved in defense responses, such as oxidative stress-related events and the HR (Serrano et al., 2014). In our studies, the *pad4* mutant showed decreased resistance to powdery mildew, and had the largest number of fungi growth of the tested lines, while no obvious cell death was detected (**Figure 3B**). This indicated that the cell death induced by the pathogen was an active defense, rather than affecting by fungal growth. We generated *pad4* mutant plants over-expressing *VqWRKY52*, but the pathogen-induced cell death was blocked compared with the overexpression lines in WT plants (**Supplementary Figure S4**), suggesting that the function of *VqWRKY52* in pathogen-induced cell death relies on the *AtPAD4* gene in *A. thaliana*.

There are two *VqWRKY52* homologs in *A. thaliana*, *AtWRKY41* and *AtWRKY53. AtWRKY41* has been shown to be involved in seed development and defense response (Higashi et al., 2008; Zhong et al., 2014), while *AtWRKY53* mainly plays a role in leaf development, senescence and defense response (Murray et al., 2007; Ay et al., 2009; Ying and Zentgraf, 2010; Xie et al., 2011). Over-expressing *WRKY41* transgenic lines had increased resistance to *Pst*DC3000 and enhanced susceptibility to *Erwinia carotovora*, but reduced expression of *AtPDF1.2*, which was induced by MeJA (Higashi et al., 2008). *AtWRKY53* was found to promote basal resistance and *wrky53* mutants had increased susceptibility to *Pst*DC3000 inoculation (Murray et al., 2007). When we analyzed the response of the *VqWRKY52* over-expressing lines to *Pst*DC3000, similar results were obtained. The *VqWRKY52* overexpression lines showed an increased resistance to *Pst*DC3000 (**Figure 5**), and suppressed *AtPDF1.2* expression (**Figure 6**). Strong cell death induced by *Pst*DC3000 was also detected (**Figure 5C**). In contrast to *AtEDS1, AtPR1* and *AtPR5* expression following powdery mildew inoculation (**Figure 4**), expression of these genes was inhibited following *Pst*DC3000 inoculation compared with WT plants at an early stage (**Figure 6**), consistent with previous studies (Huang et al., 2016). This indicated that the cell death induced by *Pst*DC3000 and the expression of *AtPR1* and *AtPR5* in the overexpression lines are uncoupled. *PopP2* and *AvrRps4* are believed to block the functions of WRKY transcription factors, potentially, through acetylating lysine residues in the WRKY domain (Leroux et al., 2015; Sarris et al., 2015). Therefore, the inhibition of *AtPR1* and *AtPR5* post *PstDC3000* inoculation in the three overexpression lines may be affected by *PopP2* or *AvrRps4*. Alternatively, *VqWRKY52* proteins without DNA binding activity may partly interfere with regulating the expression of *AtPR1* and *AtPR5*.

We also tested the response of the *VqWRKY52* over-expressing lines to *B. cinerea*, a necrotrophic pathogen. It is known that necrotrophic pathogens have virulence strategies to promote host cell death and acquire nutrients from dead cells (Kan, 2006; Mengiste, 2012). Here, a stronger cell death was also induced by *B. cinerea* in the overexpression lines than in WT plants, and it is possible that they had an increased susceptibility, since the strong cell death (**Figure 7C**) promoted the growth of *B. cinerea.* In addition, the expression of *AtPR2* and *AtPR5* was strongly inhibited in the WT plants post inoculation compared to the transgenic lines. This suggested that overexpression of *VqWRKY52* can reduce the inhibition of *AtPR2* and *AtPR5* expression. The expression levels of *AtPR1* showed significant difference with post inoculation of *G. cichoracearum* and *Pst*DC3000 among WT and three overexpression lines (**Figure 6**). However, no significant difference was found on post *B. cinerea* inoculation (**Figure 8**). This indicated that over-expressed *VqWRKY52* can’t affect the expression of *AtPR1.* The expression levels of *AtPDF1.2* was enhanced at 24 hpi while suppressed at 48 hpi (**Figure 8**). Since *AtPDF1.2* was induced by MeJA (Higashi et al., 2008), this suggested that MeJA signaling was enhanced in an early stage. However, strong cell death facilitated the infection progress, which finally resulted in the fact that even enhanced MeJA signaling did not increase the resistance. Our results also indicated that over-expressing *VqWRKY52* increased the expression of SA signaling pathway related genes, which promote *B. cinerea* infection.

The ROS burst occurs hours after pathogen attacks, and is essential in regulating HR cell death (Mur et al., 2008). *WRKY7*, *8*, *9*, and *11* can regulate the ROS burst and cell death in *N. benthamiana* through controlling the expression of NADPH oxidase (Adachi et al., 2015). In our studies, the accumulation of O_2_^-^ and H_2_O_2_ was detected following the inoculation with three different pathogens (**Figures 3D**, **5D**, and **7D**) in the transgenic lines and WT plants, and accumulation levels correlated positively with HR related cell death. This suggested that the ROS burst may control pathogen-induced cell death in *VqWRKY52* overexpression lines.

Interestingly, leaves of 3-week-old plants in soil showed low promoter activity. This was consistent with our result (**Figure 1**). However, 2-week-old plants grown on Murashige-Skoog (MS) basal medium showed strong promoter activity. Although the age of the plants may impact the promoter activity, we suppose that the high humidity also plays important roles. High humidity often occurred together with pathogen infection and can promote pathogen growth and compromise plant disease responses (Zhou et al., 2004; Cai et al., 2015). This may suggest that *VqWRKY52* plays essential roles in plant defense. In conclusion, our results suggest that *VqWRKY52* may be involved in SA dependent signaling and pathogen-induced cell death. Future studies are needed to investigate the regulatory mechanisms of *VqWRKY52* mediated HR related cell death.

## Author Contributions

XipW and XiaW designed the study. XiaW, RG, and MT contributed to most of the experiments. XiaW and DW constructed the vectors, DW and CG performed data analysis. ZL and RW assisted with the analysis of the results. XiaW and XipW wrote the manuscript. All of the authors approved the final manuscript.

## Conflict of Interest Statement

The authors declare that the research was conducted in the absence of any commercial or financial relationships that could be construed as a potential conflict of interest.

## Footnotes

1 http://www.arabidopsis.org/index.jsp

2 http://www.genoscope.cns.fr
