# Supplementary material for: Ectopic Expression of the Wild Grape WRKY Transcription Factor VqWRKY52 in Arabidopsis thaliana Enhances Resistance to the Biotrophic Pathogen Powdery Mildew But Not to the Necrotrophic Pathogen Botrytis cinerea
Source: Front Plant Sci. 2017 Jan 31;8:97. doi: 10.3389/fpls.2017.00097 (PMC5281567; doi:10.3389/fpls.2017.00097)
Supplement: Supplementary file 1 [file Table_1.DOCX]

| Gene | Forwards primer | Reverse primer |  |
| --- | --- | --- | --- |
| *VvWRKY52* | CCTCTTGATGATGGGTTTAGTT | GTCTTCCACGGTAGGTGATTT |  |
| *VvActin1* | GATTCTGGTGATGGTGTGAGT | GACAATTTCCCGTTCAGCAGT |  |
| *AtPR-1*  *AtEDS1* | GTGGGTTAGCGAGAAGGCTA  TCATACGCAATCCAAATGTTTAC | ACTTTGGCACATCCGAGTCT  AAAAACCTCTCTTGCTCGATCAC |  |
| *AtPdf1.2* | GAAGCACAGAAGTTGTGCGA | TGTAACAACAACGGGAAAATAAACA |  |
| *AtPR2* | TCGATGAGAATAAGAAGGAACCAAC | ATAACAACATACTACACGCTGAAAG |  |
| *AtICS1* | CTTCCGTGACCTTGATCCTTTCT | CAGCGATCTTGCCATTAGGATC |  |
| *AtPR5* | GCACAGAGACACACACAAAA | TGTTCCTTAGAGTGAAGTCTG |  |
| *AtActin2* | AGTGTCTGGATCGGTGGTTC | CCCCAGCTTTTTAAGCCTTT |  |

Supplementary Table 1 Gene-specific primers used for qRT-PCR.
