# Supplementary material for: Ectopic Expression of the Wild Grape WRKY Transcription Factor VqWRKY52 in Arabidopsis thaliana Enhances Resistance to the Biotrophic Pathogen Powdery Mildew But Not to the Necrotrophic Pathogen Botrytis cinerea
Source: Front Plant Sci. 2017 Jan 31;8:97. doi: 10.3389/fpls.2017.00097 (PMC5281567; doi:10.3389/fpls.2017.00097)
Supplement: Supplementary file 2 [file Data_Sheet_1.DOCX]

**Supplementary Figure 1** The nucleotide sequence blast between *VqWRKY52* from *V. quinquangularis* cv. Shang-24 and *VvWRKY52* from *V. vinifera.*
