# Supplementary material for: Ectopic Expression of the Wild Grape WRKY Transcription Factor VqWRKY52 in Arabidopsis thaliana Enhances Resistance to the Biotrophic Pathogen Powdery Mildew But Not to the Necrotrophic Pathogen Botrytis cinerea
Source: Front Plant Sci. 2017 Jan 31;8:97. doi: 10.3389/fpls.2017.00097 (PMC5281567; doi:10.3389/fpls.2017.00097)
Supplement: Supplementary file 4 [file Data_Sheet_3.DOCX]

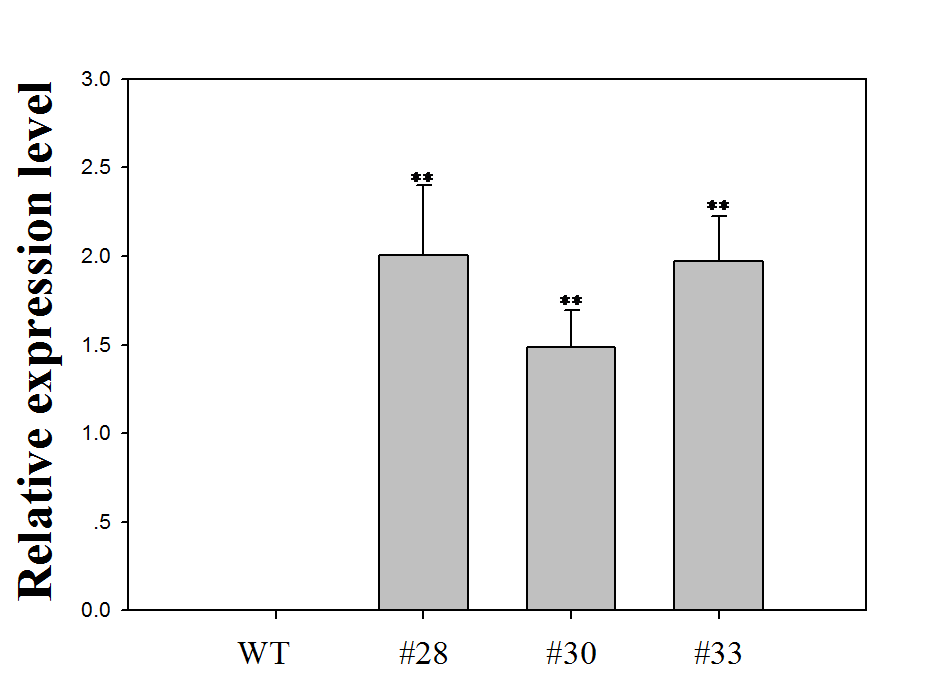


**Supplementary Figure 3** The mRNA level of *VqWRKY52* in WT and three Over-expressing lines (#28, #30, #33). Bars represent the mean ± SD from three independent experiments. Asterisks indicate statistical significance between the over-expressing lines and WT plants (** *P*<0.01, Student’s *t* test).
