# Supplementary material for: Ectopic Expression of the Wild Grape WRKY Transcription Factor VqWRKY52 in Arabidopsis thaliana Enhances Resistance to the Biotrophic Pathogen Powdery Mildew But Not to the Necrotrophic Pathogen Botrytis cinerea
Source: Front Plant Sci. 2017 Jan 31;8:97. doi: 10.3389/fpls.2017.00097 (PMC5281567; doi:10.3389/fpls.2017.00097)
Supplement: Supplementary file 5 [file Data_Sheet_4.DOCX]

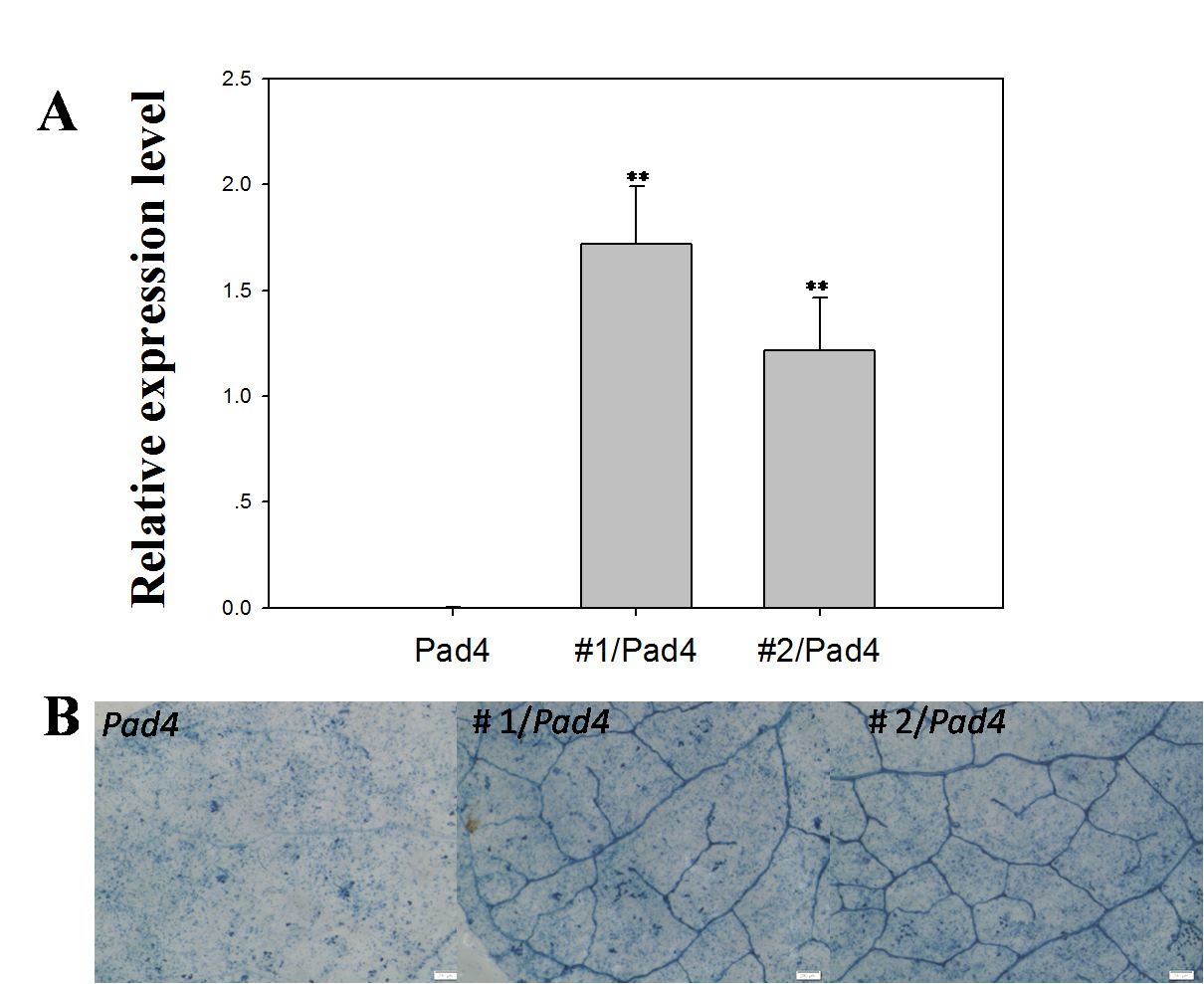


**Supplementary Figure 4** Over-expressed *VqWRKY52* in *Pad4 mutant.* (A) The mRNA level of *VqWRKY52* in *Pad4* and two Over-expressing lines (#1/ *Pad4*, #2/ *Pad4*). (B) Plant cell death were stained with trypan blue at 7 days post-inoculation. Scale bar= 200 μm. Bars represent the mean ± SD from three independent experiments. Asterisks indicate statistical significance between the over-expressing lines and WT plants (** *P*<0.01, Student’s *t* test).
